# Environmental Behavior, Sources, and Effects of Chlorinated Polycyclic Aromatic Hydrocarbons

**DOI:** 10.1100/tsw.2007.75

**Published:** 2007-03-02

**Authors:** Takeshi Ohura

**Affiliations:** Institute for Environmental Sciences, University of Shizuoka, 52-1 Yada, Shizuoka 422-8526, Japan

**Keywords:** air pollutants, chlorinated polycyclic aromatic hydrocarbons, emission source, environmental fate, human health risk, PAHs, photostability, toxicity

## Abstract

The environmental sources and behaviors of chlorinated 2- to 5-ring polycyclic aromatic hydrocarbons (ClPAHs). ClPAHs are ubiquitous contaminants found in urban air, vehicle exhaust gas, snow, tap water, and sediments. The concentrations of ClPAHs in each of these environments are generally higher than those of dioxins but markedly lower than the concentrations of the parent compounds, PAHs. Environmental data and emission sources analysis for ClPAHs reveal that the dominant process of generation is by reaction of PAHs with chlorine in pyrosynthesis. This secondary reaction process also occurs in aquatic environments. Certain ClPAHs show greater toxicity, such as mutagenicity and aryl hydrocarbon receptor activity, than their corresponding parent PAHs. Investigation of the sources and environmental behavior of ClPAHs is of great importance in the assessment of human health risks.

**ABBREVIATIONS:** chlorinated polycyclic aromatic hydrocarbons, ClPAHs; polychlorinated dibenzo-*p*-dioxins, PCDDs; polychlorinated dibenzofurans, PCDFs; polychlorinated naphthalenes, PCNs; polychlorinated biphenyls, PCBs; chlorofluorenes, ClFlu; 9-chlorophenanthrene, 9-ClPhe; 3,9-dichlorophenanthrene, 3,9-$Cl_2$Phe; 9,10-dichlorophenanthrene, 9,10-$Cl_2$Phe; 9,10-dichloroanthracene, 9,10-$Cl_2$Ant; 3-chlorofluoranthene, 3-ClFluor; 8-chlorofluoranthene, 8-ClFluor; 3,4-dichlorofluoranthene, 3,4-$Cl_2$Fluor; 3,8-dichlorofluoranthene, 3,8-$Cl_2$Fluor; 5,7-dichlorofluoranthene, 5,7-$Cl_2$Fluor; 1-chloropyrene, 1-ClPy; 4-chloropyrene, 4-ClPy; 1,3-dichloropyrene, 1,3-$Cl_2$Py; 1,6-dichloropyrene, 1,6-$Cl_2$Py; 1,8-dichloropyrene, 1,8-$Cl_2$Py; 7-chlorobenz[*a*]anthracene, 7-ClBaA; 6-chlorobenzo[*a*]pyrene, 6-ClBaP; aryl hydrocarbon receptor, AhR.

## INTRODUCTION

Organochlorine compounds, typified by polychlorinated dibenzo-*p*-dioxins (PCDDs) and polychlorinated dibenzofurans (PCDFs), are of great concern because of their toxicological effects and associated adverse health implications[1,2,3]. These compounds are derived primarily from inefficient combustion processes and occur as unwanted byproducts in the synthesis of various chlorinated products[4,5,6]. Environmental trend data on these compounds have been reviewed by Alcock and Jones[5]. Polycyclic aromatic hydrocarbons (PAHs), which are known (or in some cases suspected) to be carcinogenic, mutagenic, or both, are also generated from the combustion of organic compounds[7,8,9,10,11]. The formation mechanisms of both toxic pollutants—dioxins and PAHs—therefore have some processes in common, and chlorinated PAHs (ClPAHs) can be produced concurrently. Because, structurally, ClPAHs are hybrids of dioxins and PAHs, they are suspected of having similar toxicities. This review presents current knowledge on the environmental occurrence, sources, and biological effects of ClPAHs.

## LEVELS OF CLPAHS IN THE ENVIRONMENT

ClPAHs are a class of compounds with one or more chlorines attached to the aromatic rings of a PAH (Figure 1). In studies of ClPAHs in the environment, naphthalenes with four to eight chlorines attached—polychlorinated naphthalenes (PCNs)—have been comparatively well investigated[12,13,14,15,16,17,18]. Technical PCN mixtures known as Halowax and Nibren wax have physical and chemical properties similar to those of polychlorinated biphenyls (PCBs), which exhibit specific aryl hydrocarbon receptor (AhR) activities[12,19,20]. In addition to their emission as byproducts of product manufacture, PCNs are released to the environment from waste incineration processes, in slag residues from copper ore smelters, and from some chloro-alkali processes[21,22,23]. PCNs are also present as impurities in PCB mixtures[24].

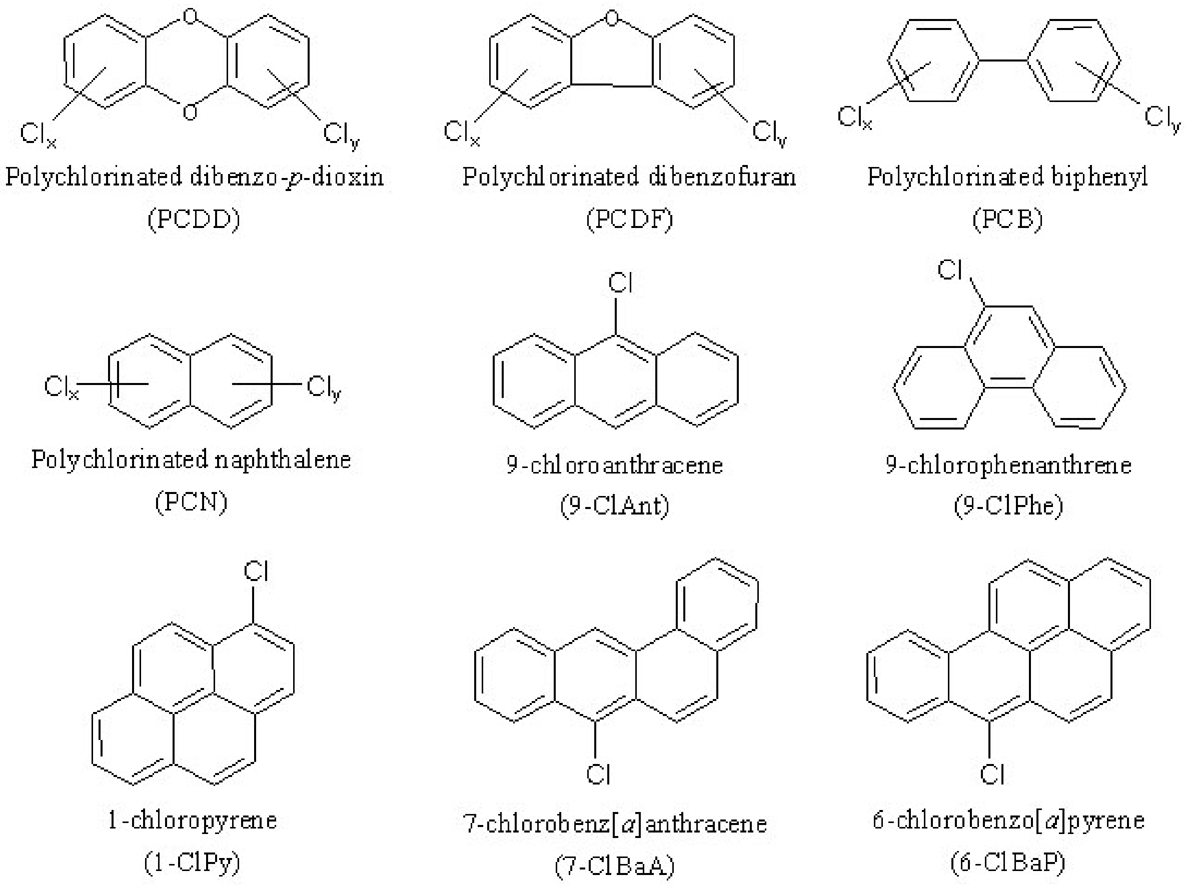

**FIGURE 1.** Structures of representative chlorinated aromatic hydrocarbons.

In contrast, because there have been only limited environmental studies of the larger (>3-ring) ClPAHs, the environmental occurrence of these compounds has not yet been fully investigated. One of the reasons is that reference substances for almost all the larger ClPAHs are not generally available commercially. Therefore, researchers who want to investigate the environmental occurrence and properties of the ClPAHs have to synthesize their own reference substances. Nevertheless, some energetic researchers have performed environmental studies of the ClPAHs. Below, I describe the environmental behavior, sources, and effects of 3- to 5-ring ClPAHs.

**Table 1.**
**ClPAH concentrations in the environment.**

| | Air ($pg/m^3$) | | | | Water (ng/L) | | Product (ng/g) | | Sediment (ng/g) | |
|---|---|---|---|---|---|---|---|---|---|---|
| ClPAH | urban street[a] | road tunnel[a] | road tunnel[b] | urban air[c] | tap water | effluent (pulp mill) | pulp | pulp sludge | settling particles[b] | sea sediment[b] |
| Cl-Flu | na | na | | na | 0.03~0.3 | nd | 0.5 | 2.0 | | |
| 9-ClPhe | na | na | | 3.5 | 0.18~0.33 | | | | | |
| 3,9-Cl3Phe | na | na | | 2.7 | 0.01~0.18 | 1.3~3.6 | 0.1~1.8 | nd~30 | | |
| 9,10-Cl2Phe | 5.6 | na | | 5.1 | | | | | | |
| 9,10-Cl2Ant | | na | | na | na | na | na | na | | |
| 3-ClFluor | 4.6 | 19.1 | | 4.5 | 0.13~0.17 | na | na | na | | |
| 8-ClFluor | 1.3 | 8.6 | | nd | | na | na | na | | |
| 1-ClPy | 11.8 | 39.6 | | 7.5 | na | na | na | na | | |
| 4-ClPy | 8.1 | 49.8 | | na | na | na | na | na | | |
| 1,3-Cl2Py | 5.6 | 4.0 | | na | na | na | na | na | | |
| 1,6-Cl2Py | 27.9 | 42.7 | | na | na | na | na | na | | |
| 1,8-Cl2Py | | | | na | na | na | na | na | | |
| 7-ClBaA | 0.4 | 2.3 | | 2.4 | na | na | na | na | | |
| 6-ClBaP | 1.9 | 6.1 | | 5.6 | na | na | na | na | | |
| ΣClPAHs | | | 3.4, 29 | | | | | | 3.2~14 | 44~1200 |
| Reference | [25] | [25] | [13] | [27] | [30] | [34, 35] | [34,35] | [34,35] | [13] | [13] |

na: not analyzed; nd: not detected.

[a] Concentrations in combined gas and particle phases.

[b] Total concentration (ΣClPAHs) shows the sum of the concentrations of chlorinated Flu, Phe, Ant, Py, and Fluor.

[c] Annual mean concentrations of ClPAH associated with particles.

## Air

A variety of the larger ClPAHs have been detected and quantified in various environmental sources, such as urban air, snow, tap water, and sediment. Their environmental concentrations are summarized in Table 1. Concentrations of ClPAHs in urban air have been investigated in a few countries. In urban air in Sweden, 9 ClPAHs with 3 to 5 rings have been detected and quantified in gaseous and particulate samples[25]. Relatively low molecular weight ClPAHs were distributed in the gaseous and particle phases, as are ambient PAHs. The concentrations combined gas and particle phase ranged from 0.4 $pg/m^3$ for 7-ClBaA to 27.9 $pg/m^3$ for 1,6- and 1,8-$Cl_2$Py (Table 1). The level of 1-ClPy detected was several hundred times lower than that of the parent PAH, Py (20 $ng/m^3$ in an urban street)[25]. ClPAH concentrations combined gas and particle phase in a road tunnel were also investigated in the same study; they ranged from 2.3 $pg/m^3$ for 7-ClBaA to 49.8 $pg/m^3$ for 4-ClPy (Table 1). Interestingly, the concentration profiles of the urban air and tunnel samples normalized against 1-ClPy were almost identical to each other. This fact indicates that the ClPAHs detected in urban air came from traffic exhaust.

The occurrences of ClPAHs in road tunnel air were also investigated in Stockholm [13]: the authors investigated simultaneously the concentrations of PCBs, PCNs, PCDD/Fs, and PAHs, as well as ClPAHs.

These substances were present in air samples at concentrations in the following order: PAHs > PCBs > PCNs > non-ortho PCBs ≅ ClPAHs > PCDD/Fs. The levels of total ClPAHs (ΣClPAHs) in tunnel air samples collected in 1991 and 1996 were 29 and 3.4 pg/$m^3$, respectively (Table 1), suggested that this decrease between 1996 and 1991 was caused by a decline in the use of leaded fuel containing halogenated scavengers (e.g., dichloroethane). Ohura et al.[26,27] synthesized 12 ClPAHs for reference and investigated the annual trends of ClPAHs in urban air particles in Shizuoka, Japan. The concentrations tended to be higher in winter than in summer, with the exception of 6-ClBaP concentrations. The annual mean concentrations were as follows: 1-ClPy (7.5 pg/$m^3$) > 6-ClBaP (5.6 pg/$m^3$) > 9,10-$Cl_2$Phe (5.1 pg/$m^3$) (Table 1). Furthermore, the temporal trends (1992~2002) of 7 ClPAHs and relationships to the parent PAHs are also recently investigated[28]. Over the study period, the concentrations of the ClPAHs, except 6-ClBaP, remained almost constant, whereas the parent PAH concentrations declined moderately. In addition there was significant correlation between the concentrations of the ClPAHs, except 3,9- and 9,10-$Cl_2$Phe, and the concentrations of the corresponding parent PAHs. This finding could indicate that the formation of ClPAHs associated with particles proceeds via that of PAHs. At the present time limited data are available on the relation ambient ClPAHs. The occurrences and profiles will need to investigate in various sites to clarify the sources and effects affecting the concentrations.

## Snow

Haglund et al.[29] identified 1-ClPy in particle fractions in snow samples collected in Sweden, but not in extracts from the aqueous phase. The (unquantified) level in the snow samples appeared to decrease with increasing distance of the sampling site from a road. These facts indicate that the possible source of the ClPAH in the snow was vehicle exhaust.

## Water

Relatively low molecular weight ClPAHs (~4-ring) have been detected in tap water[30]. The concentrations ranged from 0.01 ng/L for $Cl_2$Phe to 0.33 ng/L for 9-ClPhe (Table 1). In addition, no ClPAHs were detected in raw water samples (lake water), suggesting that the ClPAHs detected in tap water are produced by the reaction of PAHs with residual chlorine from the disinfection process. In lake and sea environments, most of the relatively high molecular weight PAHs (> 4-ring) have generally been detected in sediment samples[31,32]. PAHs present in water can be expected to pass into the sediment phase upon chlorination, but there is limited evidence that ClPAHs are detectable in sediment samples (see below).

## Pulp Mill Products

Koistinen et al.[33,34,35] investigated the concentrations of chlorinated fluorenes, phenanthrenes, alkylfluorenes, and alkylphenathrenes in effluents and pulp and biosludge from bleached kraft mills. In the effluent analysis, they detected mono- to pentachlorophenanthrenes, at maximum concentrations of 3.6 ng/L for P-ClPhe compounds, which were released into the environment at approximate levels of tens of nanograms per liter of effluent (mg/t of bleached pulp). Mono-chlorinated fluorenes and mono- and di-chlorinated phenanthrenes were also found at up to 0.5 ng/g and 1.8 ng/g, respectively, in pulp, and 2.0

ng/g and 30 ng/g, respectively, in pulp sludge (Table 1). Discharges of toxic PCDD/Fs were three orders of magnitude lower, suggesting that model substances for chloro-aromatic hydrocarbons of pulp mill origin should be prepared and their toxicity tested to evaluate possible hazards[33,34,35].

### Settling particles and sediments

Ishaq et al.[13] investigated the concentrations of ClPAHs in settling particulate matter (SPM) and sea sediments in Sweden. In the study, the levels of total ClPAHs (ΣClPAH) in the collected SPM decreased from 14 to 3.2 ng/g with increasing sampling distance from the urban center (Table 1). Also, the levels in SPM were lowest in those of other aromatic hydrocarbons measured simultaneously (PAHs, PCBs, PCNs, and PCDD/Fs). The authors suggest that the presence of ClPAHs in SPM is attributable to an urban product such as vehicle exhaust.

The levels of ΣClPAH in sea sediments ranged from 44 to 1200 ng/g (Table 1)—comparable with or higher than the corresponding levels of PCNs (22~1900 ng/g) and PCDD/Fs (4.4~170 ng/g)[13]. The sampling site where the highest level was detected was near a Mg plant, an Fe/Mn smelter, and a chlor-alkali plant. Levels of PCNs and PCDD/Fs were also markedly high at this site, suggesting that these industrial activities affect environmental levels of these halogenated compounds.

## SOURCES OF CLPAHS

In light of the production mechanisms established by studies of PAHs and dioxins, it is no wonder that the principal sources of ClPAHs in the air are traffic and incineration facilities, as well as aromatic pollutants. Dichloroethane as a scavenger contained in leaded gasoline may be the source of chlorine in ClPAHs[25,29]. Some ClPAHs have been detected in vehicle exhaust gas, although they have not been quantified[29].

Studies of the production of ClPAHs in other combustion processes have investigated emissions from the combustion of chlorine-containing materials. Wang et al.[36,37] investigated the mechanism of formation of ClPAHs in the polyvinylchloride (PVC) combustion process. Levels of naphthalene, biphenyl, Flu, Phe, Ant, Py, and Fluor and their chlorinated derivatives increased with increasing furnace temperature within the experimental range of 600–900 °C. The concentration of total ClPAHs gradually increased from 13.58 μg/g PVC at 600 °C to 101.95 μg/g PVC at 900 °C. Dichlorinated Phe was detected only at 800 °C and Ant only at 900 °C, suggesting that ClPAHs are easily formed at these higher furnace temperatures. These ClPAHs might be formed by chlorination of the parent PAH with hydrogen chloride (HCl) released from the PVC, because direct scission of PVC chains to form chlorine-containing compounds is a minor decomposition pathway.

Yoshino and Urano[38] investigated the chlorination reactions of PAHs (Ant, Py, and Fluor) with HCl or chlorine in the gas phase at high temperatures in a laboratory-scale electric furnace. The yield of chlorinated PAHs from $Cl_2$ gas was much higher than that with HCl gas. The reactions under $Cl_2$ gas were preferentially processed at 400–600 °C—a range different from the abovementioned preferential range for ClPAH production from PVC combustion. This discrepancy may be reconciled by comparing the results of a comparison of 1-ClPy concentrations in fuel — vehicle exhausts would be the principal source— and air[25]; they suggested that ClPAHs are formed not in the primary pyrosynthethic process in the cylinders, together with the PAHs, but at a later stage. Therefore, the higher temperatures at which ClPAHs are produced in combustion experiments show that ClPAHs in air are formed in secondary reactions.

Because in the experiments of Ohura et al.[26,27] the concentrations of various ClPAHs were significantly correlated with each other, these compounds might be emitted from common sources. Also, factor analysis based on the experimental data of ClPAHs and PAHs in air suggest that the emission sources for ClPAHs except for 6-ClBaP may be similar to the sources of the parent PAHs[28]. As is suggested by the above discussions, the production of ClPAHs and dioxins through the combustion of organic materials requires chlorine. The occurrence of ClPAHs and dioxins simultaneously should be investigated further to give us more information on their production processes in the environment and their possible sources.

## FORMATION OF CLPAHS

Nilsson and Colmsjö [39] confirmed that PAHs undergo chlorination in carbon tetrachloride ($CCl_4$) solution as a chlorine source under UV irradiation. Formation of ClPAHs under irradiation is enhanced compared to formation in darkness, indicating that ClPAHs are also produced by photochemical chlorination of PAHs. In addition, chlorination of PAHs adsorbed on carbon soot or urban dust particles has also been investigated under gaseous $Cl_2$ and in $CCl_4$ solution [40]. Although the reactions of PAHs adsorbed on carbon black or urban dust particles were faster than those in solution, the effect of UV irradiation on chlorination seemed minor. This was probably because adsorption onto the carbon black or urban dust particles stabilized the PAH in the face of photolytic reaction.

ClPAH formation has been confirmed in aqueous chlorination reactions[41,42,43]. In these reactions, PAHs were treated with bubbling chlorine gas or with sodium hypochlorite solution under various pH conditions. The chlorination reactions proceed under pseudo-first-order kinetics, and at $pH < 6$ they tend to produce not only chlorinated products but also oxygenated ones. In contrast, reactions at $pH > 6$ tend to produce oxygenated products as the major ones. These facts indicate that disinfection by chlorination in the treatment of water supply systems leads to the formation of ClPAHs.

Sugiyama et al.[44] investigated the chlorination of Py in soil components with sodium chloride under xenon irradiation. Chlorinated pyrenes as the reactants were produced in large amounts on metallic dioxides such as silicon and titanium dioxides under irradiation, whereas they were produced in small amounts on metallic oxides such as aluminum, magnesium, and ferric oxides, suggesting that photochemical chlorination is depended on the characterization on the metallic surface.

## FATE OF CLPAHS

Atmospheric reactions of PAHs fall into two broad categories: (i) heterogeneous processes involving particle-associated compounds (i.e., high-molecular-weight PAHs), such as photolysis/photooxidation and gas–particle interactions; and (ii) homogeneous gas-phase reactions of volatile 2- and 3-ring PAHs and semivolatile 4-ring PAHs, initiated by OH (daytime) and $NO_3$ (nighttime) radicals and ozone. These reactions lead to significant degradation of PAHs in ambient particles and the formation of products more polar than the parent PAHs. Furthermore, PAHs emitted in air, and their atmospheric reactants, are deposited on, or in, various environmental substrates such as water and soil, where these pollutants can be further transformed. Consequently, the overall picture of the environmental fate of pollutants is complex.

Ohura et al.[26,27] investigated the photostabilities of ClPAHs in an organic phase (toluene). The irradiation system simulated atmospheric photoreactions of particle-associated ClPAHs[45]. The photolysis rates of all of the ClPAHs fitted the pseudo-first-order reaction model. The ClPAHs decayed according to pseudo-first-order reaction rate kinetics, with photostabilities increasing in the order 6-Cl-BaP < 1-Cl-Py < 7-Cl-BaA < Cl-Phe < Cl-Fluor, which is consistent with the trend for the parent PAHs. However, the rate constants did not vary significantly with increasing extent of chlorination.

The photoproducts of the photolytic reactions were also tentatively identified[27]. The photolyses of ClPhe and 7-ClBaA were confirmed to proceed by initial abstraction of chlorine, followed by oxidative degradation. The oxidized byproducts of photolysis of atmospheric PAHs may be more toxic and mutagenic than their precursor PAHs[46,47]. Although it is not clear whether the oxidized byproducts of ClPAHs are ubiquitous in the environment or body tissues, the toxicities of the byproducts should also be determined.

## TOXICITY OF CLPAHS

PAHs and chlorinated aromatic hydrocarbons such as TCDD/Fs and PCBs exhibit a variety of biological activities. One of the well-established mechanisms by which (chlorinated) aromatic hydrocarbons can exert their toxic effects is via the function of the aryl hydrocarbon receptor (AhR). The AhR-mediated activities of ClPAHs have been determined by using yeast assay systems[48, 49]. Activity was observed for all the 3- to 5-ring ClPAHs tested but was at levels lower than that of 2,3,7,8-TCDD. The activities of relatively high molecular weight ClPAHs (> 4-ring) were lower than those of the parent compounds. However, for the relatively low molecular weight ClPAHs, such as chloroanthracenes and chlorophenanthrenes, activity tended to increase with increasing chlorine substitution[49].

Some studies have reported on the mutagenicity of ClPAHs. These experiments have been carried out with *Salmonella typhimurium* TA98 and TA100 in the presence or absence of S9 activation enzyme system[50,51,52,53]. ClPAHs show mutagenicity patterns different from those of their parent PAHs: ClPAHs exhibit direct-acting mutagenicity in the *Salmonella* system, whereas their parent PAHs exhibit mutagenicity only in the presence of S9. The reasons why this toxic activity occurs remain uncertain. In addition, certain ClPAHs are reported to have other toxic effects, such as tumorigenicity and oncogene activation, as reviewed by Fu et al.[54].

## CONCLUSION

Chlorinated aromatic industrial byproducts such as TCDD/Fs, PCBs, and ClPAHs have been identified as mixtures in the environment. Although the concentrations of ClPAHs in the environment are obviously lower than those of PAHs, some could be higher than those of TCDD/Fs and PCBs. To date, environmental and toxicological studies of ClPAHs have been more limited than those of other environmental toxins such as TCDD/Fs, PCBs, PAHs, and pesticides. However, the toxicity characteristics of ClPAHs appear to differ from those of TCDD/Fs and PAHs, and we should therefore be concerned about the adverse human health effects of exposure to ClPAHs. In addition, it is important that we elucidate the environmental occurrence and behavior of these compounds so that we can develop a full and accurate understanding of their toxic potential.

## ACKNOWLEDGMENTS

I thank the Japan Society for the Promotion of Science (Grant-in-aid for Young Scientists (B); No. 60315851) and the Steel Industry Foundation for the Advancement of Environmental Protection Technology (Grant No. C-31-55) for their support of this work.
